# A Sensitive Assay System To Test Antisense Oligonucleotides for Splice Suppression Therapy in the Mouse Liver

**DOI:** 10.1038/mtna.2014.44

**Published:** 2014-09-16

**Authors:** Lorena Gallego-Villar, Hiu Man Viecelli, Belén Pérez, Cary O Harding, Magdalena Ugarte, Beat Thöny, Lourdes R Desviat

**Affiliations:** 1 Centro de Biología Molecular Severo Ochoa, UAM-CSIC, Universidad Autónoma de Madrid, CIBERER, IdiPaz, Madrid, Spain; 2 Division of Metabolism, Department of Pediatrics, University of Zürich (affiliated with the Children's Research Center and the Neuroscience Center Zürich), Zürich, Switzerland; 3 Department of Molecular and Medical Genetics and Pediatrics, Oregon Health & Science University, Portland, Oregon, USA; 4 Department of Pediatrics, Oregon Health & Science University, Portland, Oregon, USA

**Keywords:** animal models, antisense oligonucleotides, exon skipping, hyperphenylalaninemia, metabolic diseases, splicing suppression, vivo-morpholino

## Abstract

We have previously demonstrated the efficacy of antisense therapy for splicing defects in cellular models of metabolic diseases, suppressing the use of cryptic splice sites or pseudoexon insertions. To date, no animal models with these defects are available. Here, we propose exon skipping of the phenylalanine hydroxylase (*Pah*) gene expressed in liver and kidney to generate systemic hyperphenylalaninemia in mice as a sensitive *in vivo* assay to test splice suppression. Systemic elevation of blood L-Phe can be quantified using tandem MS/MS. Exon 11 and/or 12 skipping for the normal *PAH* gene was validated in hepatoma cells for comparing two oligonucleotide chemistries, morpholinos and locked nucleic acids. Subsequently, Vivo-morpholinos (VMO) were tested in wild-type and in phenotypically normal *Pah*^*enu2/+*^ heterozygous mice to target exon 11 and/or 12 of the murine *Pah* gene using different VMO dosing, mode of injection and treatment regimes. Consecutive intravenous injections of VMO resulted in transient hyperphenylalaninemia correlating with complete exon skipping and absence of PAH protein and enzyme activity. Sustained effect required repeated injection of VMOs. Our results provide not only a sensitive *in vivo* assay to test for splice-modulating antisense oligonucleotides, but also a simple method to generate murine models for genetic liver diseases.

## Introduction

RNA splicing manipulation is a rapidly expanding field of research with therapeutically relevant applications in human genetic disease.^1^ Splicing is a suitable intervention point for therapy as this approach does not alter the genome and splicing defects are a common molecular etiology in many genetic diseases. Among the splice modulating therapies, one of the most promising to date involves the use of antisense oligonucleotides (AONs). These have been used to block cryptic splice sites arising from exonic or intronic mutations, to induce exon skipping to overcome nonsense or frame-shift mutations, to promote therapeutically relevant exon inclusion, or to alter exon selection to generate specific isoforms.^2^ For Duchenne muscular dystrophy (DMD), antisense oligonucleotides are currently being tested in phase 3 clinical trials in patients carrying specific deletions, with the aim of forcing exon skipping to restore the open reading frame and recover a partially functional dystrophin.^3^

One of the major initial obstacles to the successful *in vivo* application of antisense therapeutics is the inherent instability of oligo-deoxynucleotides and their interaction with RNAse H which degrades RNA bound in RNA/DNA heteroduplexes. To overcome this, different nucleotide chemistries have been developed. The most widely used for splicing manipulation are phosphorothioates with 2′-O-modifications of the ribose residue (2′-O-methyl and 2′-O-methoxyethyl), phosphordiamidate morpholinos (PMO), peptide nucleic acid, and locked nucleic acid (LNA).^4^ PMOs have a morpholino ring moiety instead of ribose and phosphorodiamidate linkages. They have no charge and do not tend to interact with other molecules, which reduces the risk of off-target effects but precludes their conjugation with delivery agents via electrostatic interaction. Moreover, efficient *in vivo* use has been documented after covalent binding to an octaguanidine dendrimer (Vivo-morpholinos (VMOs)).^5^ LNA are ribonucleotides containing a methylene bridge that connects the 2′-oxygen of the ribose with the 4′-carbon, providing them with an exceptionally high affinity for mRNA and increased stability in plasma and in cell culture medium.^6^ In addition to oligonucleotide chemistry, the clinical potential of AON treatment for splicing intervention depends on the achievement of safe and effective delivery to target tissues, biological potency and on the avoidance of unwanted side effects, all of which are being assayed in animal models as part of preclinical testing studies.^7,8,9^

We have investigated the feasibility of antisense therapy for deep intronic pseudoexon-activating mutations in different inherited metabolic diseases.^10,11^ Pseudoexons are intronic regions with apparent exonic structure having the potential for 3′ and 5′ splice site recognition but which are not normally spliced into mRNA. Many of them are derived from *Alu* or *LINE* elements and are activated by point mutations. These pseudoexon activating mutations account for 2–3% of the total alleles in several genetic diseases.^12^ In the past few years, we have demonstrated that AONs can efficiently revert pathological pseudoexon insertion in inherited metabolic diseases.^11,13,14,15,16^ Splicing correction was also achieved for exonic cryptic splice sites activated by mutations.^17^ These studies have been performed using primary patients' dermal fibroblasts or blood lymphoblasts, where AON treatment resulted in efficient splicing correction and recovery of the protein and/or enzymatic activity up to normal levels.^10^

Although these data have established the proof-of-concept of the potential of AON treatment for splicing defects in inherited metabolic diseases, methods and strategies for *in vivo* evaluation of its therapeutic efficiency are urgently needed. Ideally, animal models with analogous mutations or humanized mice with these splice defects may be generated; however this is both expensive and cumbersome, and may possibly result in lethal enzyme defects, as is the case for KO mice,^18,19^ making it difficult to investigate therapeutic approaches in them. Nonetheless, for future clinical studies, animal models are necessary tools for developing the experimental protocols for translation of therapies from bench to bedside. Here, we have studied the *in vivo* potential of AONs used in different doses and routes of delivery as splicing-directed therapy for a liver enzyme, using as model targeted exon skipping in the phenylalanine hydroxylase (*Pah*) gene, resulting in an increase of phenylalanine (L-Phe) in body fluids (also termed hyperphenylalaninemia or HPA), as occurs in phenylketonuria (PKU, MIM#261600), a well-studied metabolic disease of autosomal recessive inheritance. In the liver, PAH metabolizes L-Phe to tyrosine (L-Tyr) and its deficiency in humans leads to HPA and severe mental retardation unless adequate treatment is implemented.^20^ Several orthologous mouse models of HPA and PKU have been generated by germline mutagenesis, followed by molecular characterization.^21^ The here presented *Pah*^*enu2*^ mouse model carries a p.Phe263Ser missense mutation in the *Pah* gene. Wild-type (+/+) and heterozygous (*Pah*^*enu2*/+^) mice exhibit normal blood L-Phe concentration (<100 µmol/l) while a severe HPA phenotype (blood Phe >1,200 µmol/l) arises only in homozygous *Pah*^*enu2/enu2*^ mice.

Our working hypothesis was to use AONs in cellular and murine models to target specific exons of the endogenous *Pah* gene resulting in exon skipping of the pre-mRNA and thus producing a nonfunctional transcript. It is expected that splice suppression of the *Pah* pre-mRNA will result in a decrease in liver (and kidney) PAH activity that can be easily monitored indirectly by measuring blood L-Phe concentration, now a sensitive biomarker for the effectiveness of this antisense treatment. We have used both wild-type and *Pah*^*enu2/+*^ mice, both with normal L-Phe levels (<100 µmol/l), to test for the efficacy of exon skipping with different AON doses. Our results confirm the feasibility of antisense therapy for inborn metabolic diseases, involving liver as target organ, and providing additional evidence for the use of AONs as an antisense method to phenocopy splicing-associated diseases in animals as described.^22,23^

## Results

### Exon skipping of *PAH* pre-mRNA in hepatoma cells

**Figure 1:**
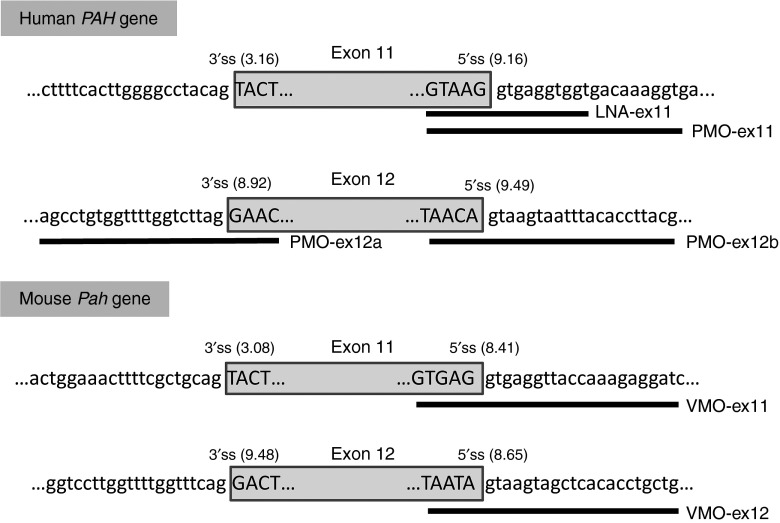
**Antisense oligonucleotides (AONs) used in this study.** The figure shows the sequences of human *PAH* and murine *Pah* gene regions corresponding to exons 11 and 12 and the bases targeted by the different AONs. Exonic sequences are typed in upper case while intronic DNA is in lower case. The extension of the various PMO, LNA and VMO sequences are indicated by the black bars. The MaxEnt (http://genes.mit.edu/burgelab/maxent/Xmaxentscan_scoreseq.html) scores for the 3′ splice site (3′ss) and 5′ splice site (5′ss) are indicated in parentheses.

The human *PAH* gene is composed of 13 constitutively spliced exons. A recent report indicated that exon 11 has a weak 3′ splice site that is vulnerable to mutations affecting splice-regulatory sequences.^24^ For antisense-mediated splicing, we thus chose to target the 5′ splice site of exon 11, and for comparison, the 3′ and 5′ splice sites of exon 12, which both have a high splicing score (**Figure 1**). In PKU patients, out-of-frame skipping of exon 11 or exon 12 resulting from well-characterized *PAH* mutations c.1066-3C>T or c.1315+1G>A, respectively, is associated with a severe HPA phenotype (see http://www.pahdb.mcgill.ca/ or http://www.biopku.org).

**Figure 2:**
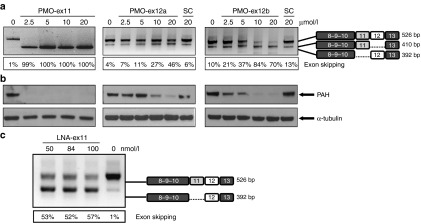
**Antisense treatment of Hep3B cells for suppression of human PAH.** Analysis of exon 11 and exon 12 splice suppression by using different concentrations of PMO-ex11, PMO-ex12a and PMO-ex12b in human Hep3B cells, are shown by (**a**) end point RT-PCR of *PAH*-mRNA, indicating the estimated percent of exon skipping and (**b**) western blot of PAH. The lower panel in (**b**) shows α-tubulin as loading control. (**c**) RT-PCR analysis in untreated cells and cells treated with different amount of LNA-ex11. The identity of the DNA-bands is shown schematically on the right. SC, scrambled oligonucleotide.

We first analyzed suppression of exon 11 and 12 splicing *in vitro* using the human hepatoma cell line Hep3B as a model. We tested two different commercially available chemistries, PMO and LNA, both having high target affinity and potent biological activity. The oligonucleotides used to target sequences in exons 11 and 12 of the human *PAH* gene are shown in **Figure 1**. Hep3B cells were transfected with different amounts of PMO or LNA (2.5–20 µmol/l) and RT-PCR for *PAH*-mRNA analysis was performed after 24 hours. As can be seen in **Figure 2a**, PMO at doses 2.5–20 µmol/l efficiently induced exon 11 skipping resulting in absence of PAH protein. Using PMO-ex11 at doses lower than 2.5 µmol/l also resulted in efficient exon skipping (**Supplementary Figure S1**). This is in agreement with previous observations in human cell lines that exon 11 is vulnerable to skipping.^24^ In fact in some experiments, some amount of exon 11 skipping was observed in untreated cells or cells treated with a scrambled oligonucleotide (**Figure 2a** and **Supplementary Figure S1**). For exon 12, only partial skipping was observed for PMO targeting the 3′ splice site. The best results were obtained using 10–20 µmol/l PMO-ex12b, targeting the 5′ splice site (**Figure 2a**). On the other hand, LNA targeting exon 11 used at the recommended nmol/l concentration range only produced partial exon skipping (**Figure 2c**), although we confirmed that cells were readily transfected (93–95%) using a fluorescent LNA (data not shown).

### Exon skipping of *Pah* pre-mRNA in mice

Our results, along with the reported risk of hepatotoxicity and lower sequence specificity for LNA,^25,26^ prompted us to focus on administering morpholino antisense oligonucleotides to mice targeting exons 11 and 12 of the *Pah* gene. To ensure efficient delivery, we used VMO, in which the morpholinos are covalently linked to an octaguanidine dendrimer allowing transport across cell membranes after systemic delivery.^27^ Although only recently described in the literature,^28^ intravenous (i.v.) injection of VMO was observed in our experiments to be associated with high toxicity (see Methods section) which limited the number of mice to a low number of animals per condition tested.

**Figure 3:**
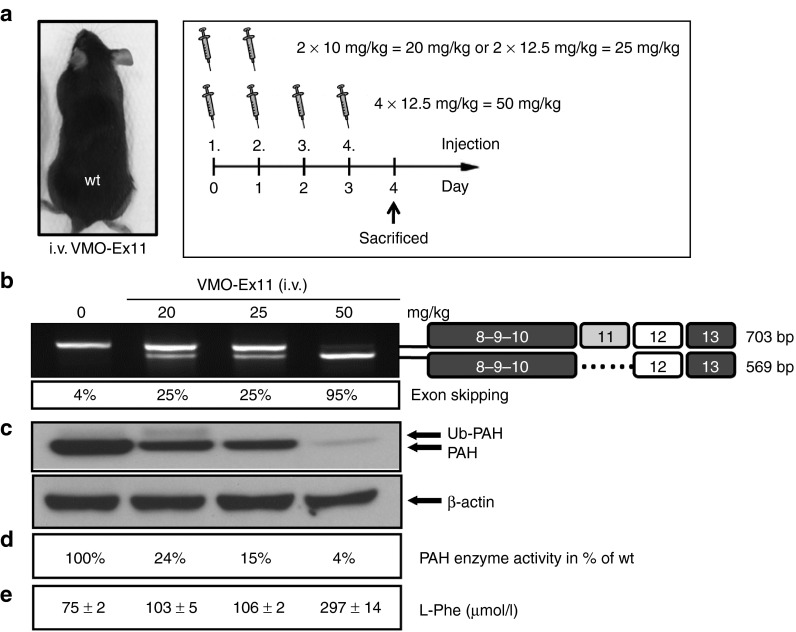
**Antisense treatment in wild-type mice leads to HPA**. Normal wild-type mice (*n* = 1) were treated with two consecutive daily i.v. injections of 10 and 12.5 mg/kg, corresponding to 20 and 25 mg/kg total dose, respectively, or with four consecutive injections of 12.5 mg/kg of VMO-ex11(total dose of 50 mg/kg). At day 4 after the first injection, mice were sacrificed for analysis, as shown schematically in (**a**). (**b**) RT-PCR analysis of *Pah*-mRNA in liver showing the identity of the bands on the right and the estimated percent of exon skipping. (**c**) Western blot analysis showing PAH protein levels in liver. Note that the faint upper band represents ubiquitinated (Ub) PAH.^45^ The lower panel shows β-actin as loading control. (**d**) PAH enzyme activity in liver extracts relative to untreated wild-type levels (=100%), and (**e**) blood L-Phe levels (mean ± SD) determined at day 4.

Initially, we tested VMO against exon 11 in wild-type mice, which were injected i.v. with 10 or 12.5 mg/kg daily doses for two or four consecutive injections, resulting in total doses of 20, 25, or 50 mg/kg (*n* = 1 per dose). All mice were euthanized and analyzed 4 days after the first injection. As can be seen in **Figure 3**, RT-PCR analysis of *Pah*-mRNA revealed that nearly complete exon 11 skipping was achieved after four consecutive i.v. injections of 12.5 mg/kg per day which is equivalent to a total of 50 mg/kg of VMO-ex11. Western blot analysis confirmed decreasing amounts of liver PAH protein while blood L-Phe levels were moderately elevated at the maximal VMO dose of 50 mg/kg (297 µmol/l; normal blood L-Phe concentration < 100 µmol/l).These results indicated that in wild-type mice repeated daily injections of relatively high amounts of VMO would be needed to achieve and maintain elevated blood L-Phe concentration, our highly sensitive biomarker for the efficiency of antisense treatment.

For all subsequent experiments, we thought to test exon skipping therapy in heterozygous *Pah*^*enu2/+*^ mice, which exhibit pre-existing partial liver PAH deficiency but retain normal blood L-Phe, as VMO treatment would have a greater likelihood of suppressing liver PAH activity to below 5% wild-type activity and may more adequately mimic the hyperphenylalaninemic phenotype of PKU. *Pah*^*enu2/+*^ mice (*n* = 1) were first treated with different doses of VMO-ex11, and blood L-Phe concentration was measured daily up to day 4 after the first injection. A single i.v. injection of 12.5 mg/kg showed no increase in blood L-Phe, correlating with residual PAH protein and activity in the range of 5–6% of normal wild-type activity (data not shown).

**Figure 4:**
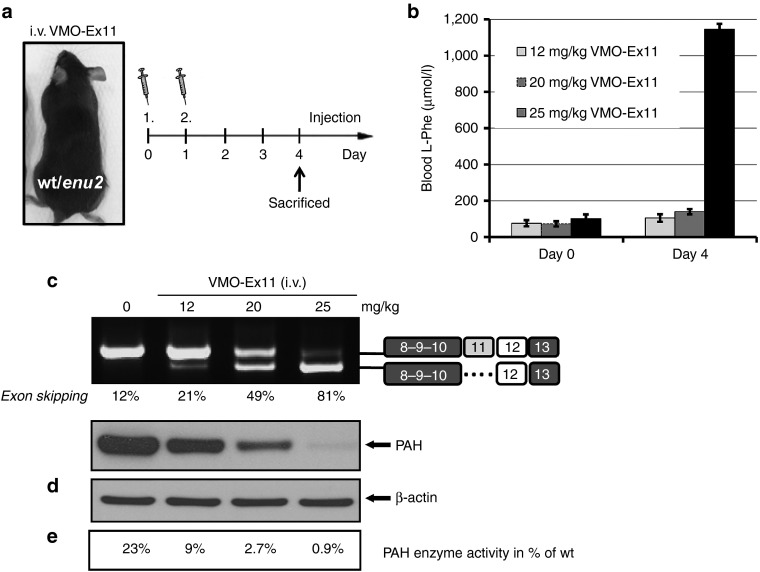
**Antisense treatment in heterozygous *Pah***^***enu2/+***^ **mice**. (**a**) Mice (*n* = 1) were treated with two consecutive i.v. injections with 2 × 6, 2 × 10, or 2 × 12.5 mg/kg of VMO-ex11 resulting in the depicted total dose of 12, 20, and 25 mg/kg, respectively, and sacrificed at day 4 after the first injection. (**b**) Blood L-Phe levels (mean ± SD), (**c**) RT-PCR analysis of *Pah*-mRNA from liver, indicating the estimated percent of exon skipping and (**d**) western blot analysis showing PAH protein levels in liver. The lower panel shows β-actin as loading control. (**e**) *PAH* enzyme activity in liver extracts relative to control wild-type levels (=100%; note that 23% of PAH activity is observed in heterozygous *Pah*^*enu2/+*^ mice).

The effect of two consecutive i.v. injections with different doses of VMO-ex11 is shown in **Figure 4**. Four days after the first injection, L-Phe levels remained at normal levels (100–140 µmol/l) at a total dose of 12 or 20 mg VMO/kg, whereas in mice treated with 25 mg/kg, L-Phe levels rose to 1,144 µmol/l (**Figure 4b**) which corresponds closely to the defined severe phenotype of HPA or phenylketonuria.^20^ The observed L-Phe levels correlated well with the results in liver analysis, where only trace amounts of normal *Pah* transcript, PAH protein and enzyme activity could be detected at the maximal dose used (**Figure 4c**–**e**). Furthermore, the effect was transient, as exon skipping levels diminished after day four and L-Phe concentration were normal from day 6 on (**Supplementary Figure S2**). Both alleles in the heterozygous *Pah*^*enu2/+*^ mice respond in the same way to VMO treatment, as sequence analysis of the normal and exon skipped band revealed that both peaks at the enu2 mutation locus (c.789T>C) are similarly represented, excluding any preferential targeting of one of the two transcripts (data not shown).

**Figure 5:**
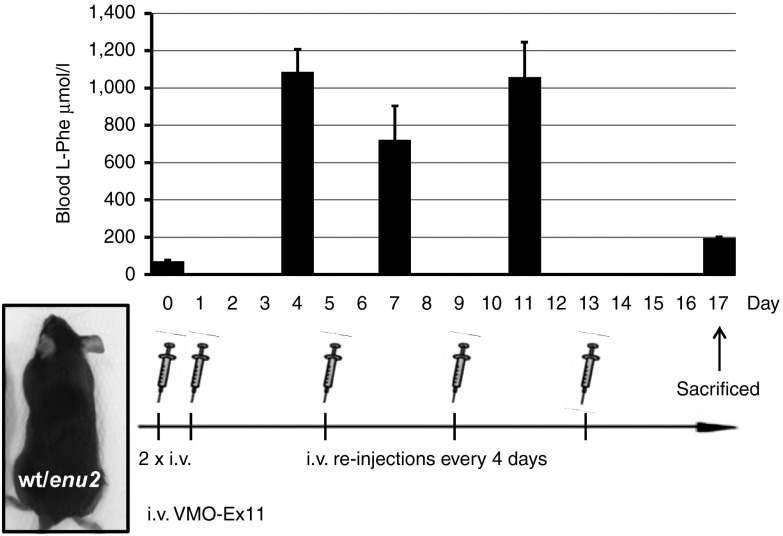
**Sustained HPA requires reinjection every four days.** A heterozygous *Pah*^*enu2/+*^ mouse was treated with two consecutive i.v. injections with 12.5 mg/kg of VMO-ex11 and subsequent reinjections at day 5, 9, and 13. Blood L-Phe levels were monitored at different time points up to day 17.

We also evaluated the dosing regime needed to maintain the HPA phenotype. Suppression of blood L-Phe clearance by VMO-induced exon skipping of *Pah* pre-mRNA depends not only on the rate of *Pah-*gene expression, but also on the turnover rates of VMO, *Pah*-mRNA and PAH protein. As depicted in **Figure 5**, we found that after injection of two initial doses of 12.5 mg/kg of VMO-ex11 into *Pah*^*enu2/+*^ mice, consecutive dosing every 4 days with 12.5 mg/kg were necessary to maintain blood L-Phe levels between 700 and 1,100 µmol/l, equivalent to a mild phenylketonuria phenotype (for a definition see).^20^

### Comparison of routes of delivery and targeting various *Pah*-exons

Using the “optimal” dosage and a regime of two consecutive 12.5 mg/kg VMO-Ex11 injections (12.5 mg VMO/kg per injection for a total of 25 mg/kg) with euthanasia and tissue analysis 4 days after initial injection, we evaluated the efficacy of intraperitoneal (i.p.) delivery of VMO. With i.p. delivery, we observed only partial exon skipping, translating into 20% residual PAH activity and L-Phe levels within the normal range (<100 µmol/l; data not shown).

**Figure 6:**
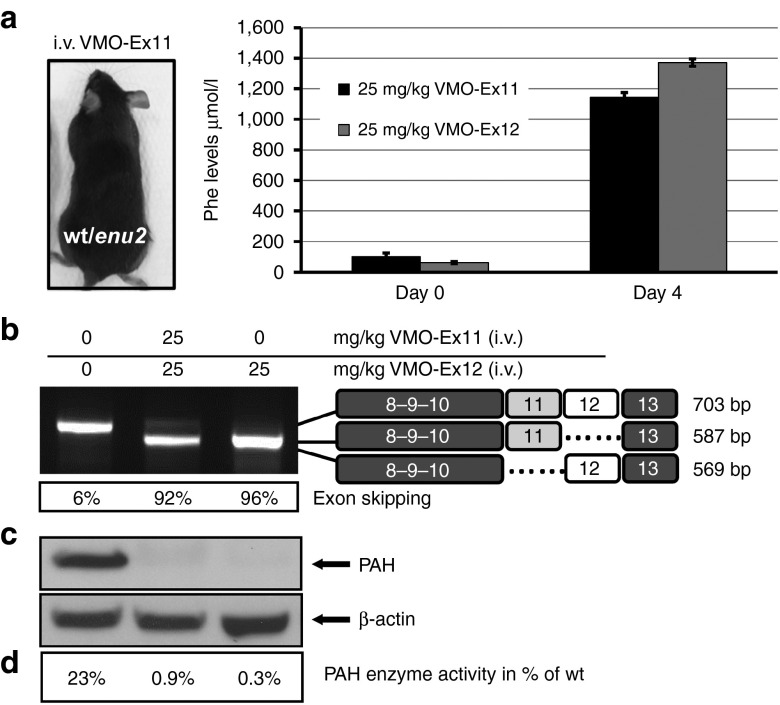
**VMO targeting exon 11 or exon 12 exert the same biological effect**. Heterozygous *Pah*^*enu2/+*^ mice (*n* = 1) were treated with two consecutive i.v. injections (of 12.5 mg/kg of VMO-ex11 or VMO-ex12) and sacrificed at day 4 after the first injection. (**a**) Analysis of blood L-Phe levels (mean ± SD). (**b**) RT-PCR analysis of *Pah*-mRNA from liver, indicating the estimated percent of exon skipping. (**c**) Western blot analysis showing PAH protein levels in liver. The lower panel shows β-actin as loading control. (**d**) PAH enzyme activity in liver extracts relative to wild-type levels (=100%).

In addition, we compared the efficiency of VMO targeting exon 12 or exon 11 delivered by i.v. injection. As shown in **Figure 6**, we obtained comparable results with either VMO-Ex11 or VMO-Ex12 including efficient exon skipping, PAH suppression, and elevated blood L-Phe concentration.

### Suppression of *Pah* expression in the kidney

**Figure 7:**
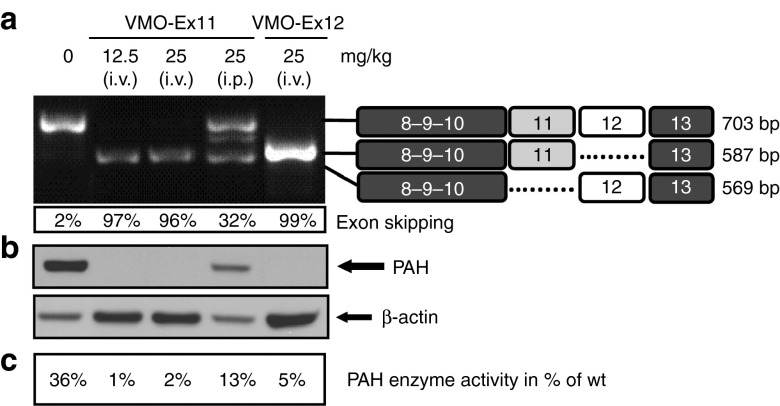
**Results in kidney from *Pah***^***enu2/+***^ **mice treated with VMO-Ex11 or VMO-Ex12.** Heterozygous *Pah*^*enu2/+*^ mice (*n* = 1) were treated with one or two consecutive injections (i.v. or i.p.) of 12.5 mg/kg of vMO-ex11 or VMO-ex12. Mice were sacrificed at day 4 after the first injection. (**a**) RT-PCR analysis of *Pah*-mRNA from kidney, indicating the estimated percent of exon skipping, and (**b**) western blot analysis showing *PAH* protein levels in kidney. The lower panel shows β-actin as loading control. (**c**) *PAH* enzyme activity in kidney extracts relative to wild-type levels (=100%).

In mice, *Pah* is mainly expressed in liver but there is also considerable expression in kidney^29^ (**Supplementary Figure S3**). We analyzed *Pah*-mRNA, as well as PAH protein and enzyme activity in *Pah*^*enu2/+*^ mice subjected to different treatment regimes. As shown in **Figure 7**, we observed equal effects whether targeting exon 11 or exon 12, and we confirmed that i.p injection of VMO is less effective than i.v. injection. Moreover, a single i.v. injection with 12.5 mg VMO/kg was sufficient to result in complete exon skipping and absence of PAH protein in kidneys of treated mice.

## Discussion

Experimental evidence in both cell and animal models has shown that antisense-mediated splicing modulation can result in therapeutically favorable transcripts for a variety of genes including dystrophin, SMN, and lamin.^1^ In this work, we provide for the first time *in vivo* evidence of the feasibility of the approach for targeting expression of a liver enzyme. For specific disease causing mutations that lead to faulty mRNA splicing, splice modulation may be used to correct the fault, as has been demonstrated through antisense treatment of patient fibroblasts.^10^ Alternatively, splice modulation can be used to model inborn errors of metabolism by interfering with native enzyme expression as we have demonstrated here. Likely disease targets include any inborn error of metabolism caused by deficiency of a hepatic enzyme, such as an aminoacidopathy, organic acidemia, or urea cycle disorder. For example, pathological pseudoexon insertions have been described in ornithine transcarbamylase deficiency,^30,31^ 6-pyruvoyl-tetrahydropterin synthase deficiency where antisense therapy efficiently suppressed pseudoexon activation,^11^ and even PKU in which a pseudoexon within the *PAH* gene, albeit of unknown clinical relevance, has recently been identified.^24^ It is assumed that the frequency of pseudoexon activating mutations is to date underestimated as transcript analysis is not usually performed for genotyping, and complete intronic sequences are not generally included in standard sequencing analysis. However, with the advent of next generation sequencing techniques deep intronic mutations will predictably be increasingly identified, as recent examples show.^32,33,34^ The approach can also be used for mutations causing activation of exonic or intronic cryptic splice sites, which are also frequent splicing defects. In this case, natural splice sites should be preserved so that blocking the cryptic splice site may effectively recover normal protein and activity.

Chemical composition of the oligonucleotide and delivery system clearly influence the biological potency and efficiency of *in vivo* antisense treatment. Both LNA and PMO/VMO have shown splice modulating activity in cells and animal models.^7,13,35,36^ The LNA oligonucleotide tested in the cellular model, although successfully transfected, was only partially effective, suggesting further sequence optimization or dosage increase may be necessary. In addition, the LNA is a 15-mer and had matches to other genomic sequences which could also contribute to its only partial effect. In contrast, the PMOs tested contained 24 nucleotides and targeted exclusively the *PAH* gene sequence. Although further studies are necessary to draw conclusions about the comparative efficiencies of the two chemistries, our aim was to achieve complete exon skipping in the *PAH* gene which was possible with PMO and VMO targeting conserved splice sites in cell culture and in mice. These results allow us to establish initial parameters for the future clinical application of splice modulation in human inborn metabolic disease. In cells, PMO resulted in efficient exon skipping at doses as low as 2.5 µmol/l for exon 11, although higher doses were needed to achieve similar exon skipping levels for exon 12. This may be attributable to the fact that exon 12 has strong splice sites, while exon 11 has a weak acceptor. Yet in mice, we did not observe any difference between VMO targeting exon 11 or exon 12 at the optimal dosage and regime tested (two consecutive i.v. injections with single doses of 12.5 mg/kg). Differences in AON uptake between cell transfection and *in vivo* delivery, genetic background or target cell type as well as species dependent pharmacokinetics, can influence splicing regulation and result in different effects *in vivo* and *in vitro*, as previously discussed.^23^

In the cellular model, a severe reduction of the amount of PAH protein was observed both with PMO-ex11 or PMO-ex12 at 20 µmol/l although at the RNA levels exon skipping was not complete with PMO-ex12. These differences may be attributable to experimental reasons related to the techniques used and the time point of analysis after transfection (24 hours for RNA, 48 hours for protein). In addition, skipping of exons 11 or 12 both produce an out-of-frame transcript but only in the case of exon 11 skipping is this transcript predicted to be subject to nonsense-mediated decay, as the premature termination codon introduced is located >50 nucleotides upstream of an exon–exon junction.^37^ Indeed, in some experiments, transcript reduction is detectable for VMO-ex11 treatment, as compared to VMO-ex12.

Although the delivery of oligonucleotides into whole organisms poses several challenges, we achieved 100% exon skipping in liver and kidney by simple tail vein injection, consistent with earlier reports.^8,38^ Expression levels of the target gene are important for dosage and regime, as deduced from the comparison of the results we obtained in liver and kidney, where lower VMO amounts are needed for exon skipping in kidney compared to liver, correlating with lower *Pah* expression levels and consequently fewer targets for exon skipping (**Supplementary Figure S3**).

It is important to work with the lowest possible effective dose to reduce the risk of off-target effects. Our model biochemical endpoint measure was the achievement of high L-Phe levels (>1,200 µmol/l). The observation that suppression of liver PAH activity to less than 5% wild-type activity is necessary to cause hyperphenylalaninemia in mice agrees with previous reports.^39,40^ Complete exon skipping needed to achieve high L-Phe levels represents the extreme situation but from a therapeutic point of view, we can speculate that much lower amounts of splice switching may be relevant for many recessive-inherited diseases.

PMOs have been used in the *mdx* mouse model of DMD with excellent body-wide distribution and effect after systemic administration and promising results have also been reported in clinical trials. In DMD, entry of naked PMO is facilitated by the leaky nature of the membrane in the dystrophic muscle fibers.^41^ In enzyme deficiencies, responsible for inborn metabolic diseases, a delivery agent is presumably needed for efficient cellular uptake. Different options include dendrimeric particles or cell penetrating peptides. The high toxicity of VMO immediately after i.v. injection was evident at the doses used (see Methods section) and surviving mice showed a decrease in the rate of weight gain. Although initial reports in the literature described no signs of toxicity with systemic VMO treatment and no immune response after repeated applications,^9^ a recently published article described the contrary with high mortality rates in treated mice, in line with our observations.^28^ The authors hypothesize that oligonucleotide hybridization resulting in an increase in cationic charge of the dendrimer moiety leads to blood clot formation which in turn induces cardiac arrest.^28^ Indeed, the histopathology studies conducted in the mice that died during VMO treatment in our work showed large blood clots in the cardiac chambers, with dilation of both atria and ventricles, pulmonary congestion and alveolar edema. This supports the idea of cardiac arrest due to VMO-related alteration of the clotting system as possible cause of death. The results clearly limit the potential of clinical application of VMO and favor future studies with other chemistries which have been shown to be well tolerated and without adverse effects. The *in vivo* assay system described in this work (targeting *Pah* exon 11 and monitoring L-Phe levels, *Pah* transcript and protein analysis) can be readily used in further studies comparing different chemistries and delivery agents available for clinical application for safe and efficient AON splice modulation.

Understanding the underlying mechanism in specific disease-causing mutations and developing drugs to correct them are a promising approach to treat genetic disorders, although rare diseases may pose difficulties for clinical application, including the high costs and the regulatory hurdles to overcome. Predictably, lessons learned from one disease can be translated to another related one. Here we report that AONs represent a powerful tool for splicing modulation for a liver enzyme in a physiological context in living mice. In addition, we show that the exon-skipping approach for constitutive exons provides versatility for the rapid and easy generation of murine models of genetic diseases. A similar approach was recently described by intracerebroventricular injection of AONs to phenocopy a more severe and adult-onset forms of spinal muscular atrophy,^22,23^ providing several advantages for therapy testing and characterization of distinctive pathological features of the different types of spinal muscular atrophy. With our results we confirm the potential of altering splicing of target genes expressed in tissues other than central nervous system to generate a disease model.

In our model, repeated injections are needed to maintain the HPA phenotype, which is probably related to shorter half-lives of *Pah-mRNA* transcript and PAH protein. In this case, a vectorised approach providing sustained AON production such as the engineered AAV vectors expressing antisense sequences linked to U7 snRNA tested in DMD^42^ will be the best option to test. The exon-skipping approach may be very useful for pathophysiologic studies modeling disease phenotypes of differing severity in addition to allowing evaluation of therapeutic effects. Antisense approaches may also be applied for better modeling of a severe form of the disease in existing model systems with a mild phenotype due to residual endogenous mRNA which can be targeted with specific AONs, or for *in vivo* study of protein isoforms by modulating skipping of alternative exons.

In summary, our work represents proof-of-concept for the *in vivo* use of antisense therapeutics for splicing defects in genetic disease, and for the potential of exon skipping in the flexible generation of animal models for such diseases.

## Materials and methods

*Cell culture and treatment.* Human hepatoma Hep3B cells were cultured in standard conditions in MEM containing 10% fetal bovine serum, 100 U/ml penicillin, 100 μg/ml streptomycin, and 2 mmol/l glutamine. One day prior to transfection, cells were seeded at 2 × 10^5^ cells/ml in six-well plates. Duplicate transfection experiments were performed using morpholino (PMO) and locked nucleic acid (LNA) oligonucleotides. PMOs against the conserved splice sites of exons 11 and 12 of the human *PAH* gene as well as a scrambled (SC) oligonucleotide for negative control, were designed, synthesized, and purified by Gene Tools (Philomath, OR). LNA were designed and produced by Exiqon (Vedbaek, Denmark). Gene Tools and Exiqon offer a design service for exon skipping oligonucleotides, for which the researcher provides the exonic and flanking intronic sequences. In all cases, the oligonucleotides designed target the conserved 3′ or 5′ splice sites. The sequences of the different antisense oligonucleotides used are shown in **Figure 1**. Endo-Porter delivery reagent was used to deliver PMO to cultured cells following the manufacturer's protocol (Gene Tools; www.gene-tools.com).^13^ For LNA, we used lipofectamine 2000 (Invitrogen, Carlsbad, CA) as transfection agent diluted in Opti-MEM I Reduced Serum Medium according to the manufacturer's protocol.

*Mice handling.* All mice used, wild-type and heterozygous (*Pah*^*enu2/+*^), were in the C57Bl/6 background and were young adult males or females (4–12 weeks old; 18–28 g). Mice were maintained on standard chow. Animal experiments were carried out in accordance with the State Veterinary Office of Zurich and Swiss law on animal protection, the Swiss Federal Act on Animal Protection (1978), and the Swiss Animal Protection Ordinance (1981). All animal studies were approved by the Cantonal Veterinary Office, Zurich, and the Cantonal Committee for Animal Experiments, Zurich. Genotyping was performed with genomic DNA isolated from mouse ear biopsies and using the DNeasy Blood & Tissue kit (Qiagen, Hombrechtikon, Switzerland). Conventional PCR was performed to amplify exon 7 of the mouse *Pah* gene (forward primer: 5′-CCTTGGGGAGTCATACCTCA-3′; reverse primer: 5′-CGGTTCAGGTGTGTACATGG-3′). The amplified DNA was digested with the restriction enzyme *Alw*26I (Thermo Fisher Scientific, St Leon-Rot, Germany) to identify the mutated *Pah*^*enu2*^ allele carrying the mutation p.Phe263Ser (c.789T>C) that generates a restriction site for *Alw*26I.^21,43^

*In vivo antisense treatment.* Antisense Vivo-morpholinos (VMO, morpholinos covalently linked to an octaguanidine moiety) targeting 5′ splice sites of exon 11 and 12 of the murine *Pah* gene were designed and supplied by Gene Tools. The sequences of the VMO used are shown in **Figure 1**. Mice were injected with different amounts of VMO from 6 to 50 mg/kg body weight, using i.p. or i.v. injections. Following the i.v. injection, Rimadyl (Pfizer) was administrated subcutaneously as a pain killer (5 mg/kg body weight). One to four mice per condition were tested in order to keep the number of animals as low as possible for protection of experimental animals. Mice were observed for ~15–20 minutes to follow their recovery. All treated mice became transiently lethargic following VMO injection. Approximately 20% of animals did not recover and perished within 12 hours following injection, which may be related to the formation of blood clots inducing cardiac arrest, as observed in histopathotogy studies postmortem and in line with previous observations.^28^

*Sample collection, preparation, and L-Phe measurement.* For analysis of blood L-Phe levels, mice were fasted between 4–6 hours before blood collection. Blood was collected from tail vein (~5 µl) or following decapitation on Guthrie filter cards and L-Phe concentration was determined using standard tandem mass spectrometry analysis as described.^44^ For RNA, protein and enzyme studies, the entire liver and both kidneys of each animal were excised immediately post euthanasia, placed in labeled cryotubes, snap-frozen in liquid nitrogen, and stored at −80 °C until processed. Frozen tissues were pulverized using screws cooled in liquid N_2_, and tissue powder was stored at −80 °C until analysis. For enzyme activity and immunoquantification, liver powder was lysed at 4 °C in homogenizing buffer (10 µl/mg tissue) (50 mmol/l Tris-HClpH 7.5, 100 mmol/l KCl, 1 mmol/l EDTA, 1 mmol/l DL-Dithiothreitol, 1 µmol/l leupeptin, 1 µmol/l pepstatin, 200 µmol/l phenylmethanesulfonyl fluoride) using Qiagen TissueLyser II (Qiagen AG). After centrifugation at 13,000 *g* at 4 °C for 30 minutes, supernatants were used to carry out PAH enzyme assays and were kept frozen at −80 °C for immunoblotting. Protein concentration was determined using Bradford reagent.

*PAH enzyme activity.* PAH enzyme activity in liver extracts was measured according to a highly sensitive and quantitative assay using isotope-dilution liquid chromatography-electrospray ionization tandem mass spectrometry (LC-ESI-MSMS) as described.^29^ Briefly, assay conditions included preincubation at 25 °C with L-Phe (1 mmol/l) for 4 minutes, then Fe(NH_4_)_2_(SO_4_)_2_ (100 ìmol/l) was added, and incubation was continued for one more min. After 5 minutes total preincubation time, BH_4_ (75 ìmol/l) was added to start the reaction. Between 2.5 and 5 µl (2.5–55 µg) of total protein lysate extracted from mouse tissue was used. Reaction time was 2 minutes. The amount of L-Tyr produced was determined by LC with ESI-MSMS. Prior to analysis, the amino acids are derivatized to propyl chloroformate derivatives, using the commercially available PhenomenexEZ:faast kit. Protein concentrations were determined using Pyrogallol Red protein dye binding assay. Specific PAH activities are expressed in nmol L-Tyr produced per minute, per mg total protein.

*RT-PCR.* For *in vitro* studies after transfection with different amounts of oligonucleotides, Hep3B cells were harvested at 24 hours and total RNA was isolated using Trizol. To amplify the human *PAH*-cDNA, total RNA was first reverse-transcribed with an oligo(dT) primer, followed by PCR amplification of the *PAH* gene region corresponding to exons 8–13 using the following primers: forward primer 5′-CATGTGCCCTTGTTTTCAG-3′ and reverse primer 5′-TTCACAGCTGACAGACCACA-3′.

For *in vivo* studies with mice, total RNA was isolated from 20 to 30 mg of mouse liver tissue using QIAmp RNA blood mini kit (Qiagen) according to the manufacturer's protocol. Random primed cDNA was prepared from 1 µg of total RNA using the Reverse Transcription kit (Promega, Wallisellen, Switzerland). PCR amplification of the region from exon 8 to exon 13 of the *Pah-mRNA* was performed using the following primers: forward primer 5′-CTAGTGCCCTTGTTTTCAGA-3′ and reverse primer 5′-AGGATCTACCACTGATGGGT-3′. Amplified products were separated by agarose gel electrophoresis and the excised bands analyzed by direct sequencing after extraction with QIAEX II Gel Extraction kit (Qiagen). The amplified PCR bands were quantified by densitometric analysis and reported as estimated percent exon skipping (relative to total amounts of amplified products in each lane).

*Western blot.* Quantification of PAH protein was performed by western blot analysis with whole mouse liver lysates or with cells harvested 48 hours after treatment. Equal amounts of lysed extracts (40 µg protein) were loaded on a 4–12% NuPAGE Novex Bis-Tris precast gel (Invitrogen). After electrophoresis, proteins were transferred to a nitrocellulose membrane (iBlot Gel Transfer Stacks, Regular) in an iBlot Gel transfer device (Invitrogen) for 7 minutes. Immunodetection was carried out using commercially available anti-PAH antibody PH8 (Abcam, Cambridge, UK) followed by a second antibody goat anti-mouse-IgG-HRP (Santa Cruz Biotechnology, CA, USA). For loading control, membranes were immunostained with either a polyclonal anti-β-actin or a monoclonal anti-α-Tubulin antibody, produced in mouse (Sigma-Aldrich, St Louis, MO A2228 and T9026, respectively). Antibody binding was detected by enhanced chemiluminescence (Amersham ECL).

**SUPPLEMENTARY MATERIAL** **Figure S1.** Antisense treatment of Hep3B cells for suppression of human PAH. **Figure S2.** Time course analysis of the effects of the antisense treatment on transcript, protein and L-Phe levels in heterozygous *Pah*^*enu*2/+^ mice. **Figure S3.** Expression levels of PAH protein in liver and kidney in heterozygous wt/enu2 mice untreated and treated with VMO-Ex11.

## Supplementary Material

Supplementary Figure S1 Antisense treatment of Hep3B cells for suppression of human PAH.

Supplementary Figure S2 Time course analysis of the effects of the antisense treatment on transcript, protein and L-Phe levels in heterozygous *Pah*^*enu*2/+^ mice.

Supplementary Figure S3 Expression levels of PAH protein in liver and kidney in heterozygous wt/enu2 mice untreated and treated with VMO-Ex11.
