# Supplementary figures and images for: A Sensitive Assay System To Test Antisense Oligonucleotides for Splice Suppression Therapy in the Mouse Liver
Source: Mol Ther Nucleic Acids. 2014 Sep 16;3(9):e193–. doi: 10.1038/mtna.2014.44 (PMC4222650; doi:10.1038/mtna.2014.44)

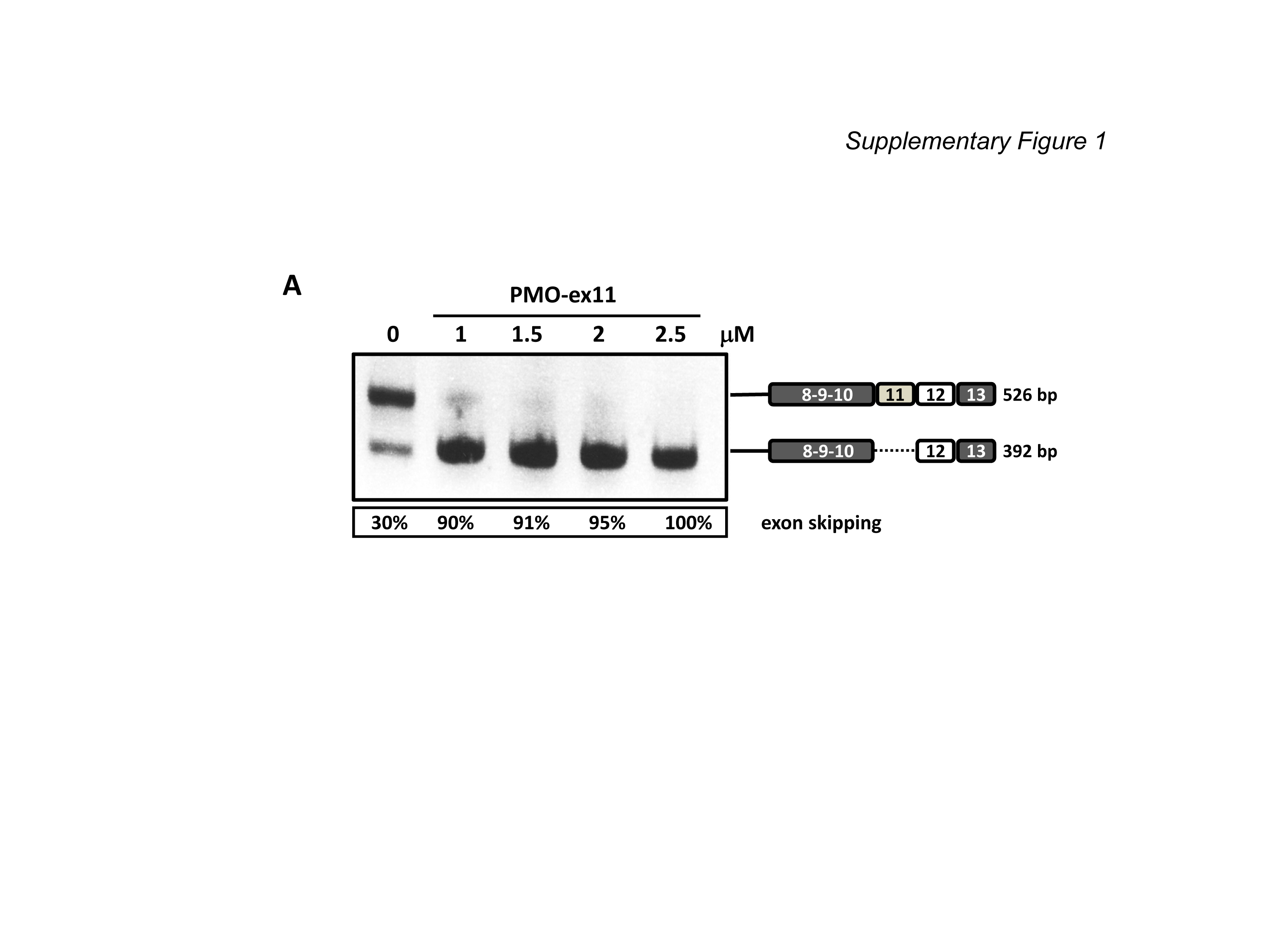

Supplement: Supplementary Figure S1 — Antisense treatment of Hep3B cells for suppression of human PAH. [file mtna201444x1.tiff]

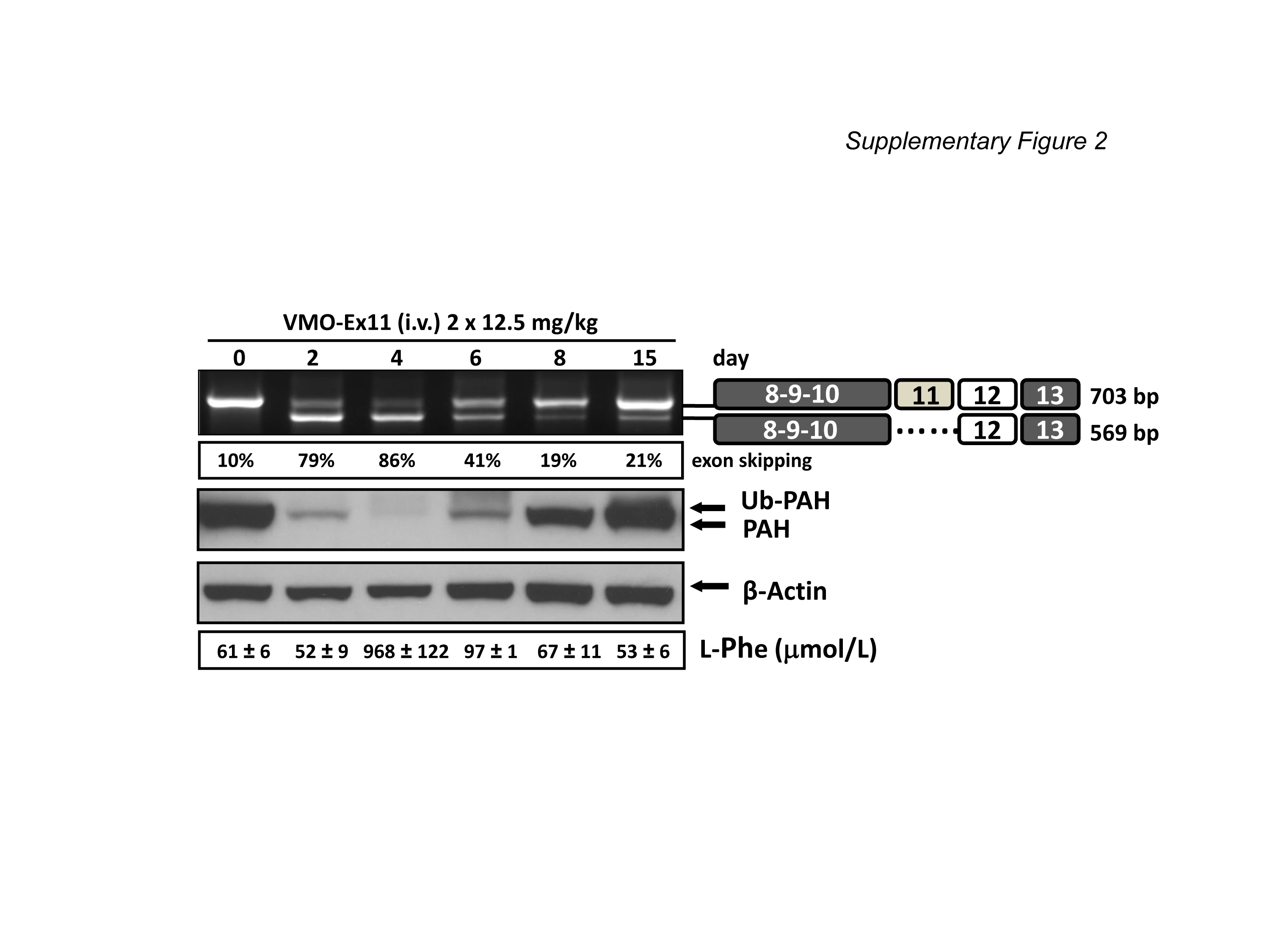

Supplement: Supplementary Figure S2 — Time course analysis of the effects of the antisense treatment on transcript, protein and L-Phe levels in heterozygous Pahenu2/+ mice. [file mtna201444x2.tiff]

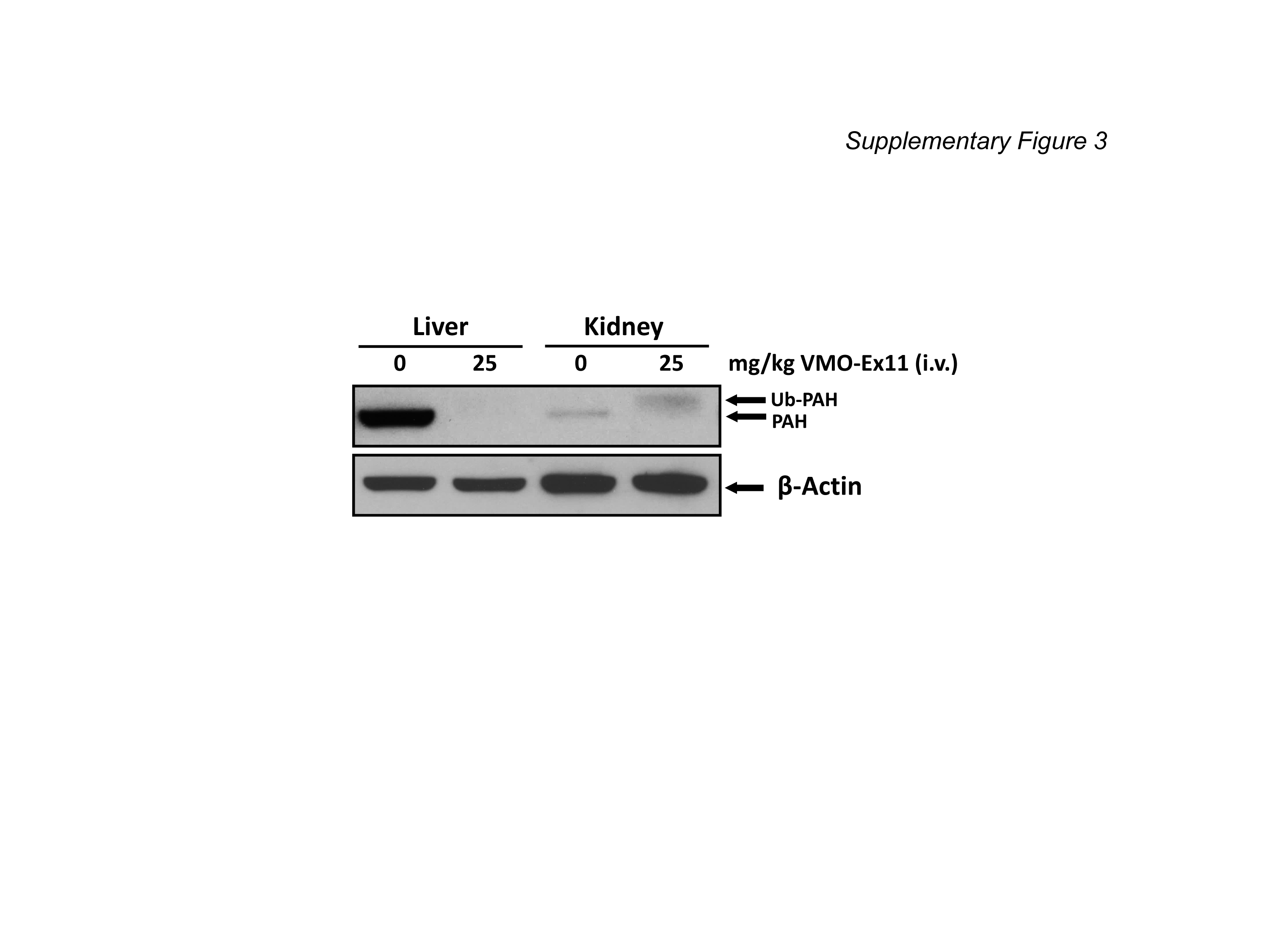

Supplement: Supplementary Figure S3 — Expression levels of PAH protein in liver and kidney in heterozygous wt/enu2 mice untreated and treated with VMO-Ex11. [file mtna201444x3.tiff]
